# Catatonia Chronicles: When the Lorazepam Challenge Shows a Delayed Response

**DOI:** 10.1155/crps/1746155

**Published:** 2025-10-09

**Authors:** Chaden Noureddine, Emil Achmad

**Affiliations:** ^1^Department of Psychiatry, Icahn School of Medicine at Mount Sinai, New York, New York, USA; ^2^Department of Psychiatry, Lenox Hill Hospital, Northwell Hospital, New York, USA

## Abstract

**Background:** Catatonia is a multifaceted disorder marked by diminished motor activity and communication, and often accompanied by heightened agitation and cognitive confusion. While historically associated with schizophrenia, recent revisions in the DSM-5 have broadened its connections to various mental and physical health disorders. The lorazepam challenge test (LCT) is used to diagnose and treat catatonia. This case challenges the traditional manifestation of catatonia and the timeline of the LCT.

**Case:** The case discussed involves a young man whose primary manifestation was cognitive impairment, ultimately diagnosed as catatonia through a traditional 2 mg LCT. Although his initial response to the LCT was negative, a marked reduction of symptoms was observed hours later.

**Discussion:** Timely diagnosis of catatonia led to symptom improvement and reduced hospitalization. This case challenges the conventional assessment timeline and highlights the need for further understanding of catatonia's pathophysiology and treatment response. It underscores the diagnostic challenges posed by catatonia's cognitive and ambiguous presentation.

**Conclusion:** The response to lorazepam, its dosage, and timing remain enigmatic, accentuating the knowledge gap in catatonia research. Further investigations are required to unravel the intricacies of catatonia's manifestation, diagnosis, and treatment response.

## 1. Introduction

Catatonia is a disorder characterized by diminished motor activity and communication, and can be present with heightened agitation and cognitive disarray [1]. Its historical classification was primarily intertwined with schizophrenia; however, recent revisions in the DSM-5 have extended its associations to encompass a broader spectrum of mental and physical health disorders [2]. Karl Kahlbaum's 1874 categorization marked the inception of catatonia, while Emil Kraepelin and Eugen Bleuler introduced divergent interpretations [1].

The DSM-5 alterations identify catatonia independently of its previous exclusive link to psychiatric conditions [2]. Diagnostic criteria require the presence of at least three out of an array of symptoms, including stupor, waxy flexibility, and agitation [2]. While traditionally correlated with schizophrenia and mania, catatonia's manifestation is not confined to these contexts, as instances have been observed in patients with concurrent general medical disorders [1]. Timely recognition is pivotal for efficacious intervention, given that catatonia may be masked by various medical conditions, delaying appropriate treatment [1]. Although the DSM-5 amendments seek to mitigate underdiagnosis, the accurate identification and management of catatonia remain intricate, particularly within populations afflicted by general medical and mental disorders [1]. Healthcare practitioners should consider catatonia as a potential diagnosis when confronted with patients meeting pertinent diagnostic criteria.

Although there is an increased interest in studying the cognitive impairment found in catatonia, the body of literature regarding this topic remains small [3]. A systematic review found that patients with catatonia tended to have worse performances in frontal and parietal cortical functions compared to healthy individuals and noncatatonic psychiatric patients [3]. Given that catatonia can present with autonomic instability, early identification and treatment are both crucial for prognosis and long-term sequelae limitation. Usually, treatment options include benzodiazepines, such as lorazepam [4]. They can be used in combination with antipsychotics, as well as electroconvulsive therapy (ECT), which involves timed electrical brain stimulation under anesthetics [4]. In recent times, new treatment modalities such as noninvasive transcranial magnetic stimulation and transcranial direct current stimulation have surfaced as well, with efficacies that are found to be comparable to those of ECT [5].

Independent of the underlying disorder, benzodiazepines, specifically lorazepam, are the mainstay treatment as there is a suggested 70%–80% remission rate of catatonia with lorazepam, which is why it is accepted as the mainstay treatment for catatonia [6]. The lorazepam challenge test (LCT) is often used to validate a catatonia diagnosis when suspected [7]. It is commonly recognized as the “Ativan challenge.” It consists of 1–2 mg of IV lorazepam, which is administered and followed by a reassessment for response 5 min after administration [7, 8]. A response is usually defined as a marked reduction of at least 50% of signs and symptoms, which are assessed using the standardized Bush Francis Rating Scale (BFCRS) [6, 7]. A recent study comparing 2 to 4 mg of lorazepam dose suggests that a 2 mg dose may be the optimal LCT dose [6].

The case presented is that of a young man who predominantly demonstrated cognitive symptoms upon presentation to the medical emergency room, and who was diagnosed with catatonia using a traditional 2 mg LCT. However, the patient's response to lorazepam was negative at first, and he only showed a marked reduction with almost complete resolution of symptoms 8 h after LCT was administered, further highlighting how little is known about catatonia and its response to benzodiazepines.

## 2. Case Description/Diagnosis

The patient was a 23-year-old man with a psychiatric history of attention-deficit/hyperactivity disorder (ADHD), no longer in treatment, had no prior psychiatric hospitalizations, and had a past medical history of COVID 12 months prior to presentation. He was domiciled with his mother, was recently employed as a doorman, and enrolled in college part-time. Patient had completed the equivalent of 2 years of university, and had no cognitive deficits at baseline, meeting all required milestones, and passing exams without issues at his university. The patient also had no known substance use history, no prior suicide attempts, no nonsuicidal self-injurious behavior, and no psychiatric records per chart search. The patient was brought by his mother to a community hospital emergency department for recent memory changes. She reports that he had returned home from work in the evening, and she noticed that he was not acting like himself as he kept repeating statements. The patient reported having no recollection of the day or of being at work. In the medical emergency department, he was also forgetting questions after several seconds. Noncontrast head computed tomography (CT) was done, and no acute changes or trauma were identified. Urine toxicology was negative. Otherwise, the laboratory results were unremarkable. The patient was transferred to a separate community hospital with inpatient psychiatric services.

Upon first contact with psychiatry, the patient was not in acute distress and was calm, cooperative, though confused. He was oriented to person and time (month, year, season); however, was unsure of the location. The patient frequently asked about his prognosis and how he was doing. He was notably ambivalent when asked about psychiatric symptoms, often sounding unsure of himself. He denied any acute changes in sleep, appetite, or energy. He denied feeling anxious or having a history of anxiety. He denied any prior panic attacks. He denied any suicidal ideation or homicidal ideation. He denied any current or past auditory or visual hallucinations. A cognitive examination showed that the patient was not able to draw a clock correctly, was not able to spell 'world' in backward order, and was not able to do serial sevens.

On the psychiatric unit, the patient was observed to not remember short-term information, asking the nursing staff the same questions, though long-term memory and answers to questions relating to identity were grossly intact. His main concerns were his memory issues, and he was not able to specify the length or timeline of symptoms. Neurology was consulted, and a magnetic resonance imaging (MRI) scan and electroencephalogram (EEG) were within normal limits. He was able to recall his major life events, some vague past events, and who his friends and cousins are, but was unable to recall details such as what he did the day prior. Further collateral was obtained from the patient's mother, who reported that the patient had been struggling with organizing his thoughts and articulating them in a cohesive manner. The patient had also been terminated from employment at two different places of employment due to performance issues. The patient was noted not to eat or drink while on the unit, which is when a potential diagnosis of catatonia was suspected.

A BFCRS was conducted and was positive for immobility, staring, mutism, verbigeration, and minimal oral intake, totaling a score of nine. A 2 mg oral LCT was administered in the afternoon with no response five and 15 min later. The next morning, 14 h later, the patient was noted to be eating and started remembering the events leading to admission. Repeat BFCRS was scored at four, which was positive for immobility and minimal oral intake. A repeat 2 mg intramuscular LCT was initiated over 1 min, with a repeat score of 0, 15 min after the second LCT was administered.

The patient's presentation was most consistent with catatonia and an underlying brief psychotic disorder. The patient was started on lorazepam 1.0 mg every 8 h and risperidone 0.5 mg every night, which was uptitrated to 3 mg every night. The patient's BFCRS remained at 0 throughout the hospital stay, and the patient was discharged with the stabilizing regimen with a plan to down-titrate lorazepam in the future. At the time of discharge, the patient's MoCA was 21/30 with deficits in attention, delayed recall, and visuospatial/executive functioning. The patient reported subjective improvement in cognition and thinking.

## 3. Discussion

The patient's presentation was ambiguous, and potential diagnoses considered by the medical team were delirium or dementia, especially considering the patient's prominent cognitive deficits. The patient's amnesia warranted a thorough neurological workup, which included a head CT, a brain MRI, and a routine EEG. The patient's catatonia symptoms could have easily been associated with other neurological and medical disorders; however, a quick assessment and diagnosis of catatonia resulted in rapid recovery of symptoms, decreased hospital stay, and avoidance of unnecessary testing and further workup. This case reflects the cognitive symptoms that may be associated with a catatonia presentation.

This case also reflects how the patient reacted to lorazepam, which ultimately led to symptom improvement and resolution by the time of discharge. However, within five to 15 min of LCT, traditionally, the patient's response to LCT would have been deemed as negative, given no change or improvement in BFCRS scoring. However, the response was still observed hours after administration of lorazepam. The half-life of lorazepam is approximated to be 10–20 h, with a reported average of 14.84 h for intravenous lorazepam [8–10]. Therefore, it would be possible for the LCT timeline to be modified depending on the metabolism and absorption of lorazepam and its metabolites. Diminished GABA-A receptor activity in the right lateral orbitofrontal and right posterior parietal cortex is believed to contribute to the impaired functioning observed in catatonia [11]. This disruption in GABAergic activity may provide an explanation for the range of motor and emotional symptoms characteristic of catatonia [1, 11]. It also clarifies the favorable response to benzodiazepines, which continue to be the primary treatment approach for this condition [1, 11].

Finally, we believe that the case reflects how little is known about catatonia and challenges the way we traditionally perceive the LCT. Some studies suggest that COVID-19 may contribute to catatonia presentation, further reflecting how little is known about this disease process [12, 13].

## 4. Conclusion

This case's ambiguous presentation prompted consideration of delirium or dementia due to noticeable cognitive deficits. Rapid diagnosis of catatonia led to symptom improvement, shorter hospitalization, and avoided unnecessary tests. This case highlights cognitive symptoms linked with catatonia and the patient's positive response to lorazepam. The delayed response to lorazepam challenges traditional assessment timelines. Further research is needed regarding the manifestation and presentation of catatonia. Additionally, this case highlights how little is known regarding the pathophysiology process behind the LCT and its timing.
